# Evaluating sampling strategies for enzootic Venezuelan equine encephalitis virus vectors in Florida and Panama

**DOI:** 10.1371/journal.pntd.0010329

**Published:** 2022-04-13

**Authors:** Kristin E. Sloyer, Mileyka Santos, Eddier Rivera, Lawrence E. Reeves, Jean Paul Carrera, Amy Y. Vittor, Anayansi Valderrama, Nathan D. Burkett-Cadena

**Affiliations:** 1 University of Florida IFAS, Florida Medical Entomology Laboratory, Vero Beach, Florida, United States of America; 2 Gorgas Memorial Institute for Health Studies, Panama City, Panama; 3 Department of Zoology, University of Oxford, Oxford, United Kingdom; 4 Emerging Pathogens Institute, University of Florida, Gainesville, Florida, United States of America; 5 Division of Infectious Disease and Global Medicine, University of Florida, Gainesville, Florida, United States of America; INDEPENDENT RESEARCHER, UNITED STATES

## Abstract

Determining effective sampling methods for mosquitoes are among the first objectives in elucidating transmission cycles of vector-borne zoonotic disease, as the effectiveness of sampling methods can differ based on species, location, and physiological state. The Spissipes section of the subgenus *Melanoconion* of *Culex* represents an understudied group of mosquitoes which transmit Venezuelan equine encephalitis viruses (VEEV) in the Western Hemisphere. The objective of this study was to determine effective collection methods that target both blood-engorged and non-engorged females of the Spissipes section of *Culex* subgenus *Melanoconion* to test the hypothesis that favorable trapping methods differ between species and by physiological status within a species. Mosquitoes were collected using two commercially available traps, (CDC-light trap and BG-Sentinel trap), two novel passive traps (a novel mosquito drift fence and pop-up resting shelters), and two novel aspirators, (a small-diameter aspirator and a large-diameter aspirator) in Darién, Panama, and Florida, USA. The total number of female mosquitoes collected for each species was compared using rarefaction curves and diversity metrics. We also compared the utility of each trap for collecting total females and blood-engorged females of four Spissipes section mosquito species in Florida and Darién. In Darién, it was found that both blood-engorged and unfed females of *Cx*. *pedroi* were most effectively collected using the mosquito drift fence at 57.6% and 61.7% respectively. In contrast, the most unfed *Cx*. *spissipes* were collected using the mosquito drift fence (40.7%) while blood-engorged females were collected effectively by pop-up resting shelters (42.3%). In Florida, the best sampling technique for the collection of blood-engorged *Cx*. *panocossa* was the large diameter aspirator at 41.9%, while the best trap for collecting *Cx*. *cedecei* was the pop-up resting shelter at 45.9%. For unfed female Spissipes section mosquitoes in Florida, the CDC light trap with CO_2_ collected 84.5% and 98.3% of *Cx*. *cedecei* and *Cx*. *panocossa* respectively in Florida. Rarefaction analysis, and both the Shannon and Simpsons diversity indices all demonstrated that the mosquito drift fence was capable of collecting the greatest diversity of mosquito species regardless of location. The finding that the proportions of unfed and blood-engorged mosquitoes collected by traps differed both among and between species has implications for how studies of VEEV vectors will be carried out in future investigations. In Florida a combination of pop-up resting shelters and use of a large-diameter aspirator would be optimal for the collection of both VEEV vectors for host-use studies. Results demonstrate that traps can be constructed from common materials to collect mosquitoes for VEEV vector studies and could be assessed for their utilization in vectors of other systems as well. Unfortunately, no single method was effective for capturing all species and physiological states, highlighting a particular need for assessing trap utility for target species of a study.

## Author summary

Venezuelan equine encephalitis virus is a potentially deadly human pathogen that is transmitted by an understudied group of tropical mosquitoes (Spissipes section of the *Culex* subgenus *Melanoconion*). These mosquitoes reside in swamps and jungles, and are challenging to identify, so studying their biology and importance in transmitting VEEV has been neglected. To further our understanding of VEEV, we compared six novel and commercially available traps to determine which traps are best for capturing these species in Panama and Florida. We found that several different types of traps are effective for collecting blood-engorged females of different species of VEEV vectors, and that traps utilized for collecting unfed specimens are not necessarily the same traps one should use for collecting blood-engorged females of the same species. Results of this study will enable researchers to better capture these important disease vectors, particularly those that are blood-engorged, which will allow researchers to determine host associations necessary for understanding VEEV transmission. This information can be used to make decisions on controlling vector species.

## Introduction

Sampling vector species is an important component of elucidating the transmission cycles of vector-borne zoonotic disease and incriminating vector species [1,2]. Because mosquito sampling strategies can be selective not only in the species trapped, but also in physiological states of the specimens (i.e., blood-engorged, or host-seeking), it is sometimes necessary to elucidate effective sampling strategies which can best be used to answer questions about vector species [1]. The Spissipes section of the subgenus *Melanoconion* of *Culex* represents an understudied group of 22 mosquito species which transmit enzootic subtypes of Venezuelan equine encephalitis viruses (VEEV) in the Western Hemisphere [3]. As a result, few studies report comparisons of specific trapping techniques for the various species and physiological statuses within species of this medically important group. These limitations make it difficult to incriminate enzootic vectors of VEEV, which can compromise the ability of vector control units to reduce vector density through targeted interventions.

The VEEV complex consists of six antigenic viral subtypes, nine species, and between one and five antigenic varieties, which are restricted to the Western Hemisphere [4,5]. Subtype I consists of varieties A/B, C, D, E, and F. Subtype I varieties A/B and C are referred to as “epizootic” strains as they are only isolated during epizootics and epidemics involving equine and human illness. In contrast, subtype I varieties D, E, and F are referred to as “enzootic” strains as they primarily occur in sylvatic transmission and are generally avirulent to equines, although subtype IE has resulted in two separate equine epizootics in Mexico in the 1990s [6]. Subtypes II-VI are also considered enzootic strains and are often referred to by common names including Everglades virus (EVEV) (VEEV-II), Mucambo (VEEV-III), Tonate (VEEV-IIIB), Pixuna (VEEV-IV), Cabassou (VEEV-V), and Rio Negro (VEEV-VI) [7].

Overwhelmingly, enzootic VEEV subtypes are transmitted by species of the Spissipes section of the subgenus *Melanoconion* of *Culex* [8]. Several species of this section are confirmed or suspected vectors of enzootic VEEV, including *Culex vomerifer* Komp, *Culex pedroi* Sirivanakarn and Belkin, *Culex adamesi* Sirivanakarn & Galindo, *Culex delpontei* Duret, *Culex ocossa* Dyar & Knab, *Culex panocossa* Dyar, *Culex spissipes* (Theobald), *Culex taeniopus* Dyar & Knab, *Culex cedecei* Stone and Hair, *Culex portesi* Senevet & Abonnenc, and *Culex gnomatos* Sallum, Hutchings, & Ferreira [9–16], a member of the vomerifer group that is morphologically similar to *Cx*. *vomerifer*, but not known to occur in Panama. Most species in this section occur in Central and South America, and the Caribbean. The only exceptions include *Cx*. *cedecei*, which is endemic to Florida, USA, and *Cx*. *panocossa*, a vector of VEEV-ID, which has become established in peninsular Florida, USA [17].

Relatively little literature is published on methods for efficiently sampling Spissipes section mosquitoes despite their importance as vectors of medically important pathogens. Sweep netting, CDC light traps, encephalitis vector survey traps (EVS), malaise traps, Shannon traps, battery-powered aspirators, and Trinidad no. 17 traps, have been used to study the biology and ecology of Spissipes section species in Panama [18–23]. Animal-baited traps, in which vertebrates are caged or confined to attract host-seeking mosquitoes, have been effective for sampling Spissipes section *Melanoconion* such as *Cx*. *pedroi* and *Cx*. *taeniopus* at enzootic VEEV foci in Central and South America [9,18,24,25]. While animal-baited traps may be effective for initial incrimination of vector species attracted to rodent hosts (the amplifying hosts of enzootic VEEV [26]), they only collect those species with host affinities for the bait taxon, and under sample other vectors such as *Cx*. *panocossa*, *Cx*. *ocossa*, and *Cx*. *spissipes* [19,21,27]. In addition, animal-baited traps typically collect low numbers, typically insufficient for virus detection via pool screening [9]. Although aspirators have been used in both Florida and Panama to collect blood-engorged females of some Spissipes section members from their resting sites [22,28,29], quantitative analysis of aspirator effectiveness is lacking. *Culex cedecei* is effectively collected by resting shelters [29] and light traps [23]. *Culex panocossa* was sampled in large numbers using CO_2_-baited CDC light traps, but blood-engorged females of this species were not captured in resting shelters [17]. This further illustrates the need for research in this area, as host-use studies will be necessary to determine the feeding patterns of *Cx*. *panocossa* in its introduced range.

Trap comparison studies to improve sampling techniques for disease vectors are important for some aspects of the vector incrimination process including surveillance for virus-positive mosquitoes and collection of blood-engorged females for blood meal analysis to determine association with vertebrate hosts. Currently, efficient methods for sampling members of the Spissipes section of *Melanoconion* have not yet been identified. Species from this group are important vectors in the transmission cycles of enzootic VEEV subtypes, including VEEV-ID which is a progenitor to epidemic VEEV-IC [30]. For this study, we evaluated six collection methods consisting of both passive (pop-up resting shelters and the mosquito drift fence) and active traps (CDC light trap, BG-Sentinel) and aspirators (small-diameter aspirator and large-diameter aspirator) for their utility in collecting mosquito species from enzootic VEEV foci in both Darién Province, Panama, and Florida, USA. We used chi-squared analysis, rarefaction, bipartite analysis, and diversity indices to quantify and compare the numbers, physiological state, and diversity of mosquitoes collected. Results of this study will allow researchers investigating the transmission of enzootic VEEV in Florida and Panama to employ effective methods of collecting a diversity of physiological states and species among the Spissipes section.

## Materials and methods

### Study sites

**Fig 1:**
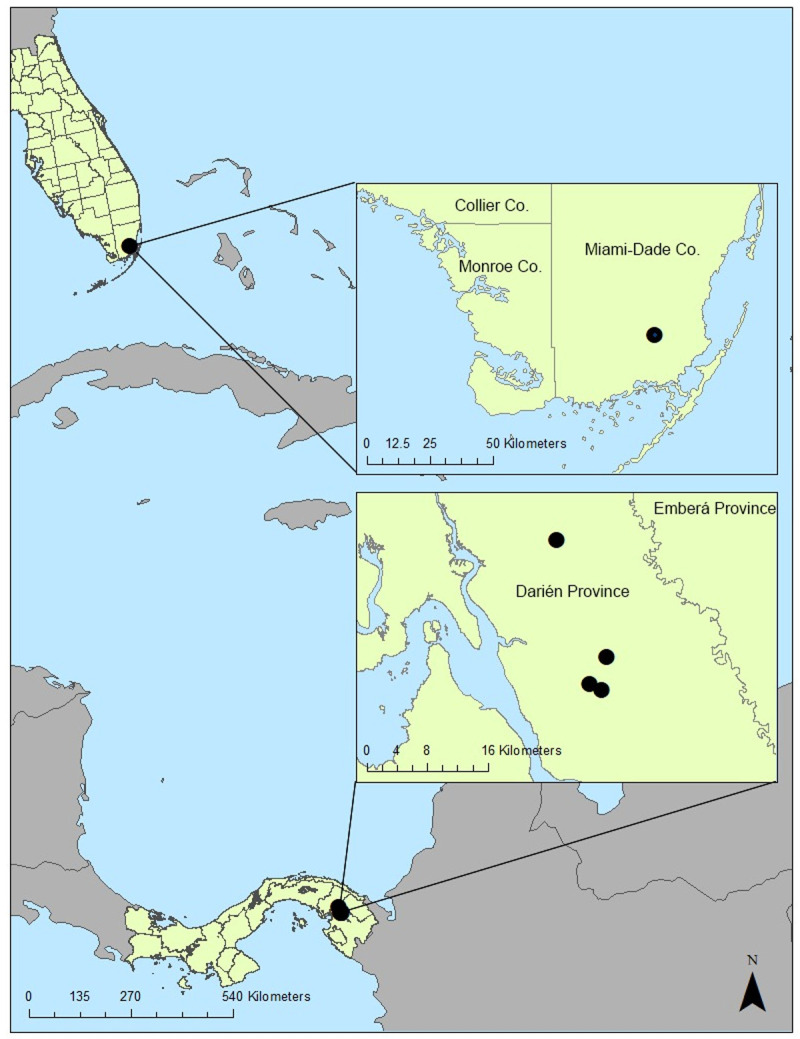
Map of the field sites in Florida City, FL, USA (1), and Metetí, Darien Province, Panama (3). Base layer country maps downloaded from: https://www.diva-gis.org/gdata

The study sites include two foci for the transmission of VEEV-ID and EVEV in rural Metetí, Darién, Panama and Florida City, Florida, respectively. The study site in Panama is a site of long-term, multidisciplinary research utilized by The Gorgas Institute in Panama City, Panama. Field comparisons of sampling techniques took place at three separate locations in an agricultural area outside of the city of Metetí in Darién Province (Fig 1). Darién Province borders Colombia and the landscape is comprised of tropical rainforest and agricultural activities where VEEV infections in humans, horses, and other domestic and wild animals have been detected [18,31]. The three locations in Metetí where trap comparisons took place were part of an agricultural community comprising cattle pasture interspersed with gallery forests, where sampling activities were focused. The primary study site in Florida was in Florida City (Miami-Dade County), approximately 10 km east of Everglades National Park and 5 km north of natural habitats that make up the Southern Glades Wildlife and Environmental Area (25.411377, -80.493494) (Fig 1). The site is near (~1km) a suburban neighborhood and is surrounded by tropical plant farms interspersed with plots of natural and invasive vegetation. This site was chosen due to the number of *Culex* (*Melanoconion*) species present, including the only two members of the Spissipes section found in the USA, *Cx*. *cedecei* and *Cx*. *panocossa*.

### Collection methods

**Fig 2:**
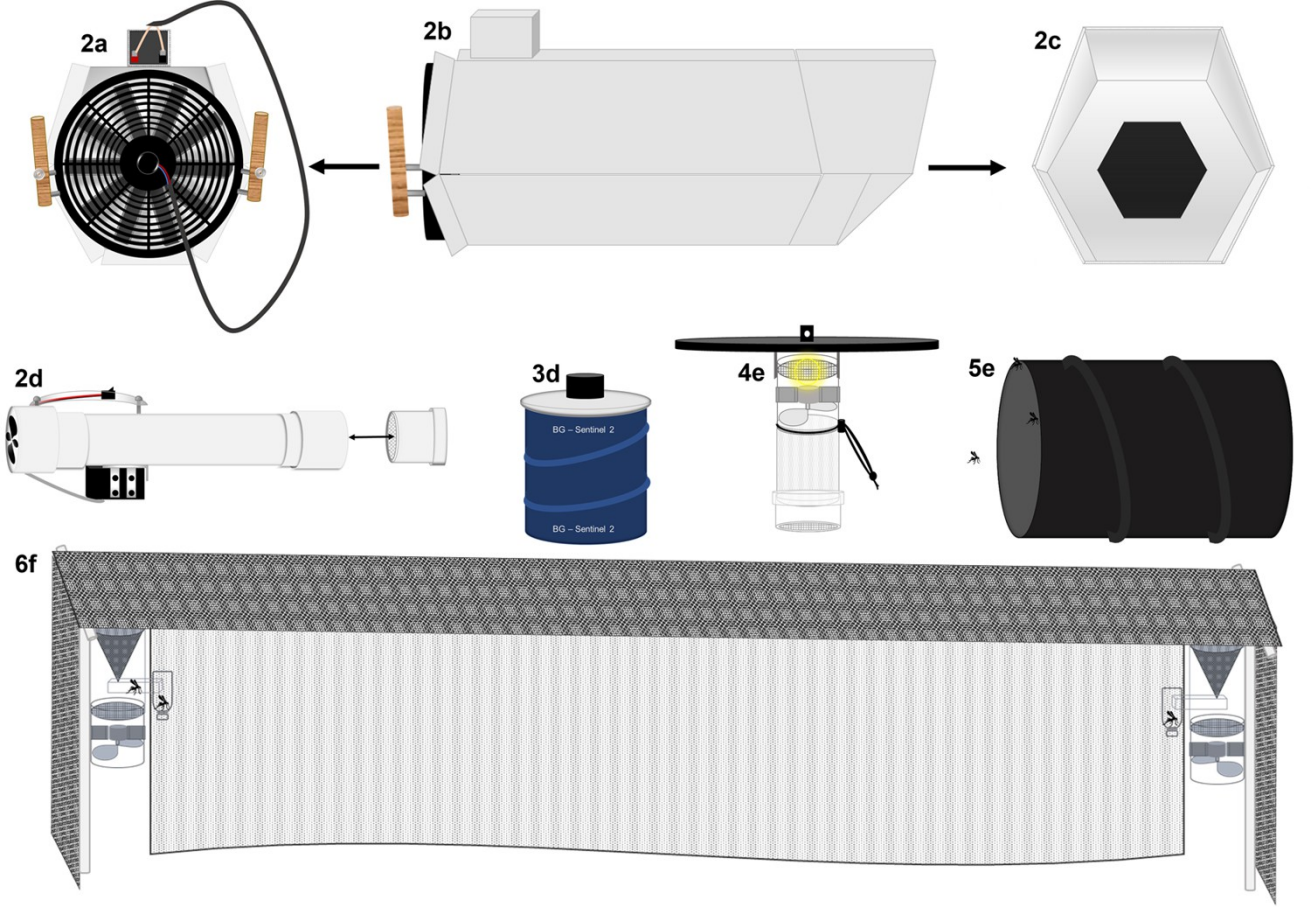
Illustrations of the six mosquito collection methods used by this study. Large-diameter aspirator is depicted in Fig 2A–2C. Lateral view of the small-diameter aspirator illustrated in 2d. BG-Sentinel and the CDC-light trap are illustrated in 2e and 2f respectively. The pop-up resting shelter and mosquito drift fence are illustrated in 2g-2h.

We evaluated diverse sampling methods to quantify their effectiveness in collecting mosquitoes, focusing on members of the Spissipes section. Sampling methods consisted of commercially available traps, novel traps, and aspirators (hereafter, all referred to as “traps”). Traps included in this study were (1) the mini-CDC light trap with incandescent light (CDC) from BioQuip Products Inc. (Model #: 2836BQ) (Rancho Dominguez, CA, USA); (2) the BG-Sentinel trap (BGS) with octanol from BioGents (Regensburg, Germany); (3) Pop-up resting shelters (PRS) [29]; (4) a novel “mosquito drift fence” (MDF) for collecting mosquitoes as they travel through the environment; (5) a large-diameter aspirator (LDA) for collecting mosquitoes from the forest floor; and (6) a small-diameter aspirator (SDA) for sampling cavity-type natural resting sites of mosquitoes (Figs 2 and S1). The novel traps (4–6) are described in detail, below.

The PRS are constructed of the stripped spring-steel coil of a collapsible laundry hamper (Walmart, Arkansas, USA) attached to a black contractor trash bag using black duct tape as described in Burkett-Cadena et al. [29]. Each PRS was set the night prior to collections on the ground with the opening facing west (away from the rising sun), and out of direct sunlight to provide a dark, cool place for mosquitoes to rest. Mosquitoes were sampled each morning approximately one hour after sunrise using a circular lid with a collection chamber which is placed over the opening of the PRS and manually pumped by hand (see Appendix A in Burkett-Cadena et al. [29]).

The MDF is an intercept trap based upon the concept of herpetological drift fences used to capture reptiles and amphibians [32] by placing a vertical physical barrier in the environment which interrupts natural movements of animals, forcing them to encounter traps at either side of the barrier. For capturing mosquitoes, dark gray vinyl window screen was used as the vertical barrier with white bridal veil fabric as an upper horizontal barrier (Fig 2H). Battery powered suction traps were placed at both ends of the vertical barrier. With this design, a mosquito that encounters the vertical barrier screen should fly upward, encounter the horizontal barrier, and follow it in either direction until it encounters one of two suction traps at either side of the drift-fence. The approximate rectangular dimensions of the vertical barrier are 3 m long and 0.75 m tall. Passive suction traps are particularly useful since they are not actively attracting one physiological state over another, they may collect a more representative cross section of a mosquito population, and are therefore less likely than baited traps to be biased toward one species or another or toward certain physiological states [1]. However, the vertical barrier of this particular design may be too short to adequately collect mosquito species which fly at higher heights, particularly in tropical forests where mosquito communities may be vertically stratified.

The LDA is a novel design, based upon a model described by Nasci [33], which was itself based upon a prototype designed by George O’Meara. The fan of the aspirator is a Mishimoto 14” slim electric radiator fan (Model: MMFAN-14) (New Castle, DE, USA) and is powered by a rechargeable 12V/6AHr gel-sealed battery. The body of the aspirator is made with white corrugated plastic (Coroplast) folded into a hexagonal shape and connected by duct tape. The dimensions are 91.4 cm on top, 76.2 cm on the bottom, to allow for a longer ‘lip’ to disturb mosquitoes resting in vegetation and leaf litter. The opening is hexagonal in shape and measures 38.1 cm from top side to bottom, and each of the six sides are equal in length and are 22.9 cm. The catch bag, a no-see-um head net (The Ultimate Survival Gear, Columbia, MO, USA), is secured to the 19.05 cm intake opening using six small binder clips (Fig 2C).

The SDA is made with a small 12V 0.92A computer fan by NMB (Model #: 09225VA-12Q-AL). The fan is encased at the large end of a polyvinyl chloride (PVC) pipe coupler and attached to a piece of PVC pipe 10.1 cm in diameter and 61 cm long. Attached to the end of the aspirator is a pipe-coupler allowing for the insertion of a collecting cup (Model #: 2846D, BioQuip Products, Rancho Dominguez, CA, USA) (Fig 2). The SDA is powered by a 6V 5Ah battery (Duracell Ultra, Bethel, Connecticut, USA), providing approximately 30 minutes of continuous run-time.

### Sampling

**Fig 3:**
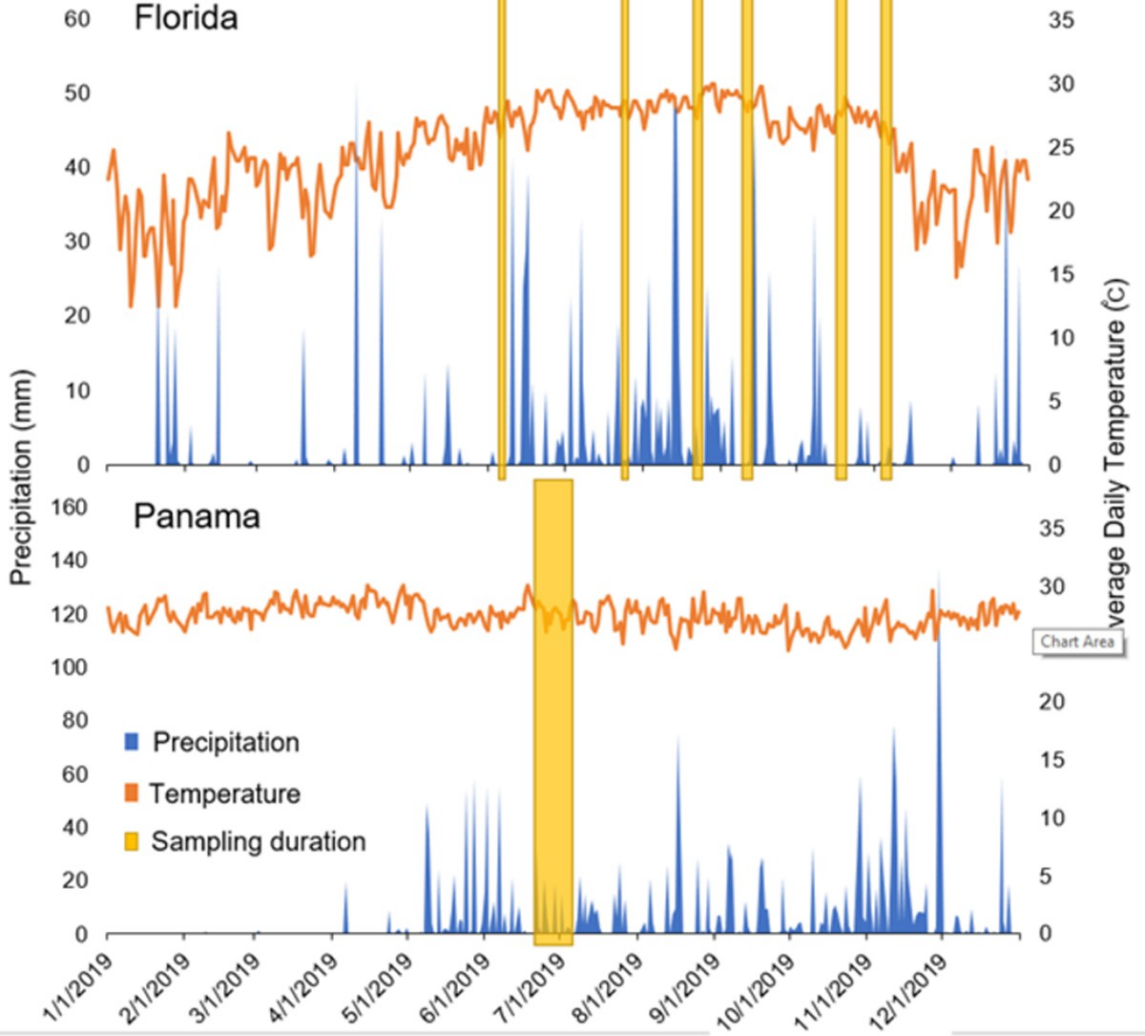
Weather data during 2019 for Florida and Panama, including precipitation (blue) and temperature (orange). Mosquito sampling periods are highlighted in yellow Panama data is from ETESA and Florida data is from NOAA.

Each sampling replicate in Panama consisted of one MDF, one BGS with octanol lure, one CDC trap baited with octanol, and 5–10 PRSs. One 3-minute aspiration was performed per replicate with the SDA and LDA in vegetation near the trapping sites on each morning of trap retrieval. Sampling in Panama was conducted between 25 June and 2 July, 2019, with one replicate occurring each night of the study for a total of eight trap comparison replicates (Fig 3). Three sampling replicates were performed in Florida each night, in total consisting of three drift fences, three BG-sentinel traps with octanol lure, three CDC light traps baited with CO_2_ (dry ice), and nine pop-up resting shelters. On each morning of trap retrieval, three aspirations each for the SDA and LDA were performed in wooded areas containing leaf-litter, and small shrubs such as naturalized arrowhead plant (*Syngonium podophyllum*), and various fern species near each of the trapping sites for three minutes on the morning of trap retrieval. Field collections at this site took place over a longer span and occurred for 3–4 nights per month with three replicates occurring each night between June and December 2019 for a total of 75 trap comparison replicates (Fig 3).

### Mosquito identification

Mosquitoes were primarily identified by external morphology using a variety of resources [34,35]. Florida mosquitoes belonging to the *Melanoconion* subgenus which were not identifiable using external morphology were identified by internal morphology of the cibarial armature using the key to the female cibarial armature of the *Culex* (*Melanoconion*) in Williams and Savage [36]. The three most common *Melanoconion* species which required identification through dissection of cibarial armature were *Cx*. *cedecei*, *Cx*. *atratus*, and *Cx*. *pilosus*. Florida CDC traps which captured more than 500 mosquitoes, were subsampled by taking 10% of the weight of the full sample and identifying all specimens in the subsample then multiplying the number of specimens of each species identified in the subsample by 10. In Panama, due to time limitations, cryptic *Melanoconion* species were taken to the lowest taxonomic level possible based on morphology. Females of the Spissipes section in Panama were identified using morphological keys [3]. Other mosquito species in Panama were identified with taxonomic keys specific to each group [3,37–49].

### Statistical analysis

A combination of statistical analyses and diversity measures were used to assess the best traps for measuring physiological state among collected mosquitoes, and diversity of mosquitoes collected. To compare the effectiveness of collecting Spissipes section species we compared the relative proportions of both unfed and blood-engorged females collected by the six trap types used in this study. A chi-squared analysis was carried out (v15.0; Microsoft Corporation) to test for the independence of distributions of unfed and blood-engorged females collected by each trap for each of the four collected Spissipes section species separately. Differences in trap efficacy between the major subgenera of *Culex* were compared by generating a bipartite graph using the R package ‘bipartite’ (R version 3.6.1) [50,51]. Sample-based diversity accumulation curves and rarefaction analysis built in the R package iNext to estimate the expected species richness trapped beyond the data provided were used to compare mosquito community metrics [50,52,53]. Finally, species richness, Simpson’s index, Shannon’s index, and Pielou’s index were calculated to compare the completeness of the community of mosquitoes sampled by each trap (v15.0; Microsoft Corporation).

## Results

**Fig 4:**
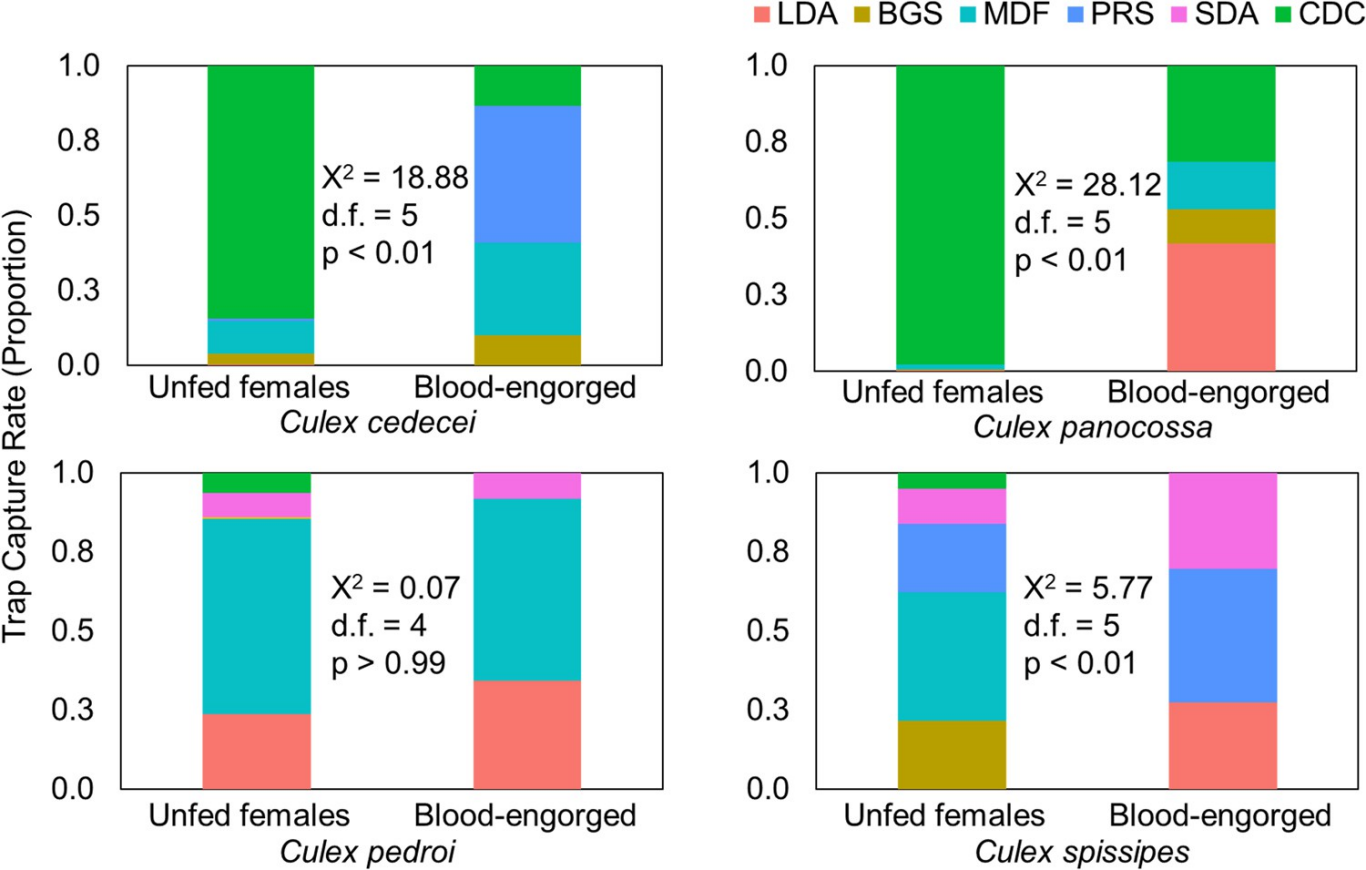
Relative proportions of unfed and blood-engorged Spissipes section mosquitoes. Mosquitoes were collected using the large diameter aspirator (LDA), BG-Sentinel (BGS), CDC miniature light trap (CDC), mosquito drift fence (MDF), pop-up resting shelter (PRS), and the small diameter aspirator (SDA) in Darién, Panama, and Florida City, FL. Chi-squared values represent differences in trap efficacy between unfed and blood-engorged of the same species.

We collected approximately 172,340 mosquitoes, representing around 71 species. Among these, we collected 57,273 Spissipes section species including *Cx*. *cedecei* and *Cx*. *panocossa* in Florida, and *Cx*. *pedroi*, *Cx*. *spissipes*, *Cx*. *vomerifer*, and *Cx*. *ocossa* in Panama. *Culex gnomatos*, a member of the vomerifer group that is morphologically similar to *Cx*. *vomerifer*, is not known to occur in Panama. Trapping techniques generally differed in effectiveness across species and physiological states for each Spissipes section mosquito. Traps that captured the majority of unfed females captured relatively few blood-engorged females of *Cx*. *cedecei*, *Cx*. *panocossa*, and *Cx*. *spissipes* (Fig 4). The distributions of unfed and blood-engorged females were significantly different (p<0.05) across trap types for these three species (Fig 4). For example, the CDC trap captured the majority of unfed *Cx*. *cedecei* (84.5%; X^2^ = 18.878, df = 5, p = 0.001) and *Cx*. *panocossa* (98.3%; X^2^ = 28.117, df = 5, p<0.001) but captured far fewer fractions of blood-engorged females of these two species (8.8% of *Cx*. *cedecei*, 23.3% of *Cx*. *panocossa*). Only *Cx*. *pedroi* was found to be sampled equally by the various trap types, with respect to blood-engorged and unfed females (X^2^ = 0.072, df = 4, p = 0.999). The PRS collected the highest percentage of blood-engorged *Cx*. *cedecei* (45.9%) but did not collect any blood-engorged *Cx*. *panocossa*. Instead, the LDA collected the greatest percentage of blood-engorged *Cx*. *panocossa* (41.9%) (Fig 4). The MDF collected the greatest percentages of unfed *Cx*. *pedroi* (61.7%) and *Cx*. *spissipes* (40.7%). The MDF also captured the highest percentage of blood-engorged *Cx*. *pedroi* (57.6%) while it did not capture any blood-engorged *Cx*. *spissipes*. In contrast, the PRS collected the highest percentage of blood-engorged *Cx*. *spissipes* (42.3%; X^2^ = 5.772, df = 5, p = 0.005) but did not collect blood-engorged *Cx*. *pedroi* (Fig 4).

**Fig 5:**
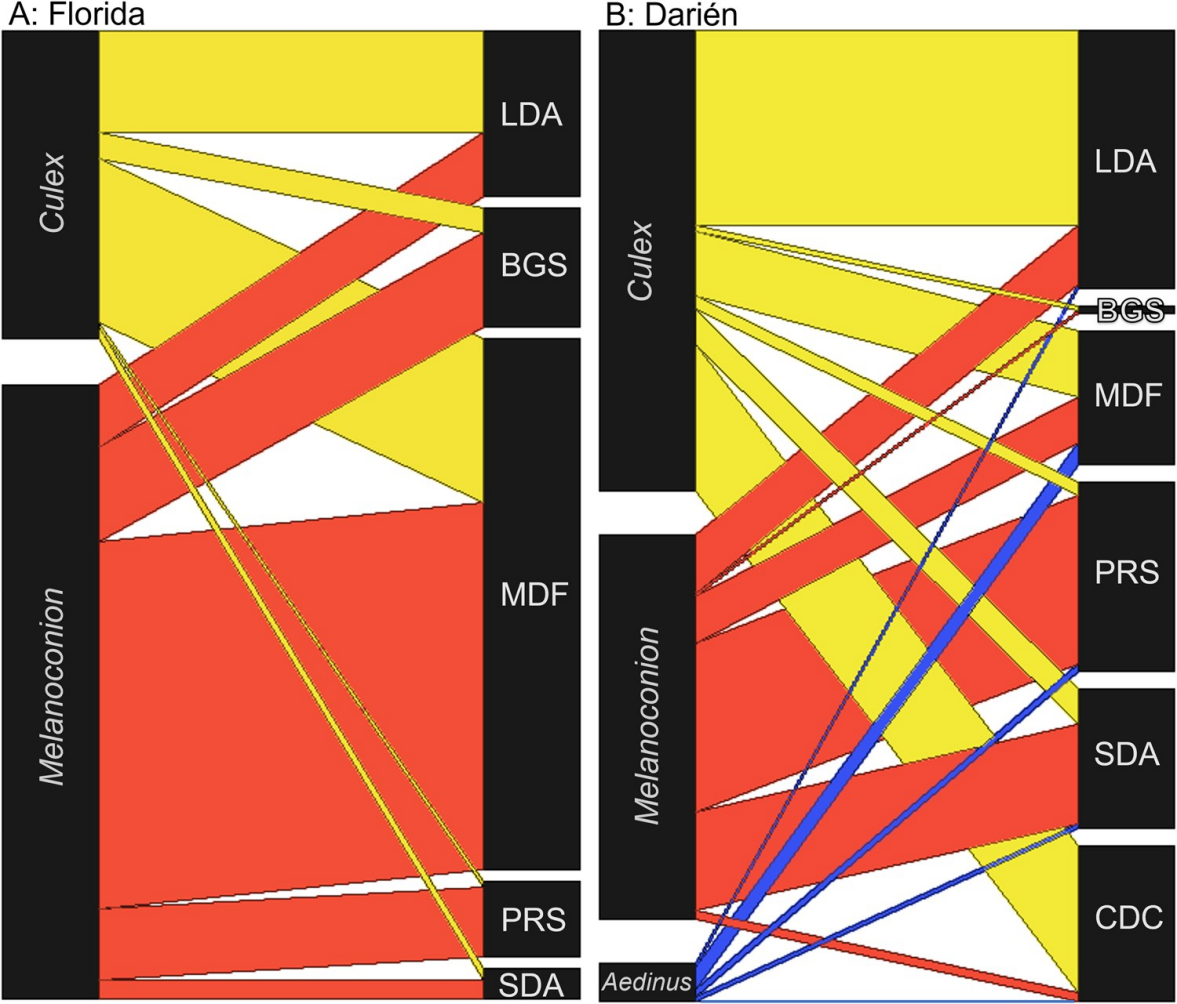
Bipartite analysis of the subgenera of *Culex* collected by each trap type. Proportions of major subgenera *Culex* (yellow), *Melanoconion* (red), and *Aedinus* (blue) captured by five of the large-diameter aspirator (LDA), BG-Sentinel (BGS), CDC miniature light trap (CDC) (shown for Panama only), mosquito drift fence (MDF), pop-up resting shelter (PRS), and the small-diameter aspirator (SDA) (excluding the CDC trap in Florida only) are depicted for Florida City, FL (A), and Darién, Panama collections (B).

In Florida, *Culex* subgenera *Melanoconion* and *Culex* were the predominant subgenera collected during the study, with *Melanoconion* representing a greater proportion captured by five of the six traps (Table 1 and Fig 5A). The only trap for which species of *Culex* (*Culex*) represented a greater proportion of the collection was the LDA (Fig 5A). *Culex* (*Culex*) mosquitoes also made up a large proportion of mosquitoes collected by the MDF, though *Culex* (*Melanoconion*) spp. still made up the greatest proportion of the collections. *Culex* (*Melanoconion*) spp. made up the highest proportions of the collections made by the BGS, PRS, and the SDA (Fig 5A).

**Table 1:** Number of each mosquito species captured for the large diameter aspirator (LDA), BG-Sentinel (BGS), CDC miniature light trap (CDC), mosquito drift fence (MDF), pop-up resting shelter (PRS), and the small diameter aspirator (SDA) in Florida City, Florida.

	Large-Diameter Aspirator (LDA)	BG-Sentinel (BGS)	CDC miniature light trap (CDC)	Mosquito Drift Fence (MDF)	Pop-up Resting Shelter (PRS)	Small-Diameter Aspirator (SDA)
*Ad*. *squamipennis*	3	1	132	3	1	0
*Ae*. *atlanticus*	32	125	554	111	1	1
*Ae*. *aegypti*	0	0	21	1	3	0
*Ae*. *albopictus*	0	1	0	2	0	0
*Ae*. *condolescens*	1	0	10	0	0	0
*Ae*. *pertinax*	948	1,426	3,632	883	8	49
*Ae*. *scapularis*	2	1	10	0	0	0
*Ae*. *taeniorhynchus*	101	7,263	5,046	896	23	19
*Ae*. *tortilis*	6	3	70	3	0	0
*An*. *crucians* complex	5	353	3,807	74	10	1
*An*. *quadrimaculatus*	5	79	924	69	239	15
*Cq*. *perturbans*	0	0	70	0	0	0
*Cx*. *(Melanoconion) atratus*	139	13	240	122	348	96
*Cx*. *(Culex) bahamensis*	1	0	0	0	0	0
*Cx*. *(Melanoconion) cedecei*	64	802	15,218	2,841	186	14
*Cx*. *(Culex) coronator*	3	31	898	9	0	0
*Cx*. *(Culex) declarator*	0	0	20	3	0	0
*Cx*. *(Melanoconion) erraticus*	106	512	26,078	1,361	376	40
*Cx*. *(Culex) interrogator*	0	1	14	4	0	0
*Cx*. *(Melanoconion) iolambdis*	0	0	0	0	3	0
*Cx*. (unknown)	5	1	90	45	0	1
*Cx*. *(Culex) nigripalpus*	1,504	344	27,005	2,419	71	173
*Cx*. *(Melanoconion) panocossa*	299	57	36,334	833	4	12
*Cx*. *(Melanoconion) pilosus*	327	13	80	309	130	110
*Cx*. *(Culex) quinquefasciatus*	0	1	10	3	0	0
*Cx*. *(Culex) salinarius*	0	0	0	5	0	0
*Ma*. *dyari*	43	222	13,606	289	9	2
*Ma*. *titillans*	17	119	2,650	64	1	0
*Ps*. *columbiae*	27	53	1,030	97	0	0
*Ur*. *lowii*	586	20	485	293	0	2
*Ur*. *sapphirina*	313	2	783	58	1	11
*Wy*. *vanduzeei*	0	0	20	1	0	0
**Totals**	**4,537**	**11,443**	**138,837**	**10,798**	**1,414**	**546**

In Panama, *Culex* subgenera *Melanoconion* and *Culex* were more frequently collected by traps than *Culex* (*Aedinus*) species, with patterns remaining relatively consistent to traps used in Florida (Table 2 and Fig 5B). *Culex* (*Culex*) was the primary *Culex* subgenus sampled by the LDA and CDC trap, and they were collected with a marginal majority by the MDF (Fig 5B). *Culex* (*Melanoconion*) made up the largest proportions of the collections made by the PRS, and the SDA (Fig 5B). A very low proportion of *Culex* (*Melanoconion*) were collected by the CDC traps baited with octanol in Panama (Fig 5B). Small numbers of *Culex* (*Aedinus*) were collected using the LDA, MDF, PRS, and the CDC trap baited with octanol, however *Aedinus* species did not comprise the greatest proportions of collections by any of the traps.

**Table 2:** Number of each mosquito species captured for the large diameter aspirator (LDA), BG-Sentinel (BGS), CDC miniature light trap (CDC), mosquito drift fence (MDF), pop-up resting shelter (PRS), and the small diameter aspirator (SDA) in Darién, Panama.

	Large-Diameter Aspirator (LDA)	BG–Sentinel (BGS)	CDC miniature light trap (CDC)	Mosquito Drift Fence (MDF)	Pop-up Resting Shelter (PRS)	Small-Diameter Aspirator (SDA)
*Ae*. *fulvus*	1	0	0	0	0	0
*Ae*. *taeniorhynchus*	1	0	0	0	0	0
*Ae*. *euplocamus*	1	0	0	0	0	0
*An*. *arribalzaga*	0	0	0	0	0	1
*An*. *pseudopunctipennis*	0	0	5	0	2	0
*An*. *punctimacula*	0	0	1	0	0	0
*Anopheles* spp.	0	1	6	0	0	0
*Cq*. *venezuelensis*	198	23	76	73	5	6
*Cx*. (*Culex*) *spp*.	99	0	175	2	0	3
*Cx*. *(Melanoconion) spp*. Atratus group	0	0	0	9	6	9
*Cx*. (*Melanoconion*) *spp*. Melanoconion group	153	0	5	49	612	335
*Cx*. *(Melanoconion) adamesi*	0	0	0	0	2	0
*Cx*. (*Aedinus*) spp.	0	0	0	0	3	0
*Cx*. *(Aedinus) amazonensis*	17	0	2	104	40	20
*Cx*. *(Anoedioporpa) browni*	0	0	0	0	2	0
*Cx*. *(Anoedioporpa) conservator*	0	0	0	0	0	1
*Cx*. *(Culex) coronator*	0	0	130	0	0	1
*Cx*. *(Culex) declarator*	0	0	4	0	0	0
*Cx*. *(Melanoconion) dunni*	0	0	9	0	0	0
*Cx*. *(Melanoconion) erraticus*	0	0	0	1	9	2
*Cx*. *(Culex) interrogator*	1	0	145	0	0	0
*Cx*. *(Culex) mollis*	1	0	0	0	0	0
*Cx*. *(Culex) nigripalpus*	643	5	247	98	50	127
*Cx*. *(Melanoconion) ocossa*	0	0	0	0	0	1
*Cx*. *(Melanoconion) pedroi*	105	0	12	79	2	35
*Cx*. *(Culex) saltanensis*	181	20	0	213	7	37
*Cx*. *(Melanoconion) spissipes*	6	12	16	83	170	87
*Cx*. *(Melanoconion) theobaldi*	16	0	0	0	2	0
*Cx*. *(Melanoconion) vomerifer*	0	0	1	0	0	0
*Li*. *durhamii*	0	0	0	8	1	5
*Li*. *flavisetosus*	0	0	0	1	0	1
*Mansonia* spp.	0	0	1	1	0	0
*Ps*. *cingulata*	29	1	25	11	1	9
*Ps*. *ferox*	2	0	0	0	0	2
*Psorophora* sp.	0	0	0	1	0	0
*Sa*. *intermedius*	0	0	0	1	0	0
*Ur*. *calosomata*	0	0	0	1	0	0
*Ur*. *ditaenionota*	0	0	0	1	0	0
*Ur*. *geometrica*	1	0	0	5	1	0
*Ur*. *incognita*	3	0	0	3	2	9
*Ur*. *lowii*	13	0	13	6	0	2
*Ur*. *nataliae*	0	0	1	1	0	0
*Ur*. *socialis*	1	0	0	0	0	0
*Ur*. *typhlosomata*	0	0	0	0	0	1
*Wyeomyia* spp.	6	2	8	8	1	1
**Totals**	**1476**	**64**	**882**	**759**	**918**	**659**

The sampled mosquito assemblages differed between Florida and Panama, affecting patterns of species diversity and evenness across traps. When considering diversity as a measure of trap effectiveness, the CDC trap (baited with CO_2_ in Florida, and with octanol in Panama), and the MDF, collected a high diversity of species in both locations (Table 3). In both Florida and Panama, the MDF captured the highest diversity of mosquito species according to both the Simpson and Shannon diversity index and had the third and second highest evenness according to Pielou’s index. The CDC trap with CO_2_ captured the second greatest diversity of mosquito species in Florida, however it had a relatively low evenness compared to the other traps. The CDC trap performed similarly in Panama despite being baited with octanol instead of CO_2_, capturing the 3^rd^ greatest diversity of species according to both the Simpson and Shannon diversity index.

**Table 3:** Values for species richness, Simpson index, Shannon index, and Pielou’s index for the large diameter aspirator (LDA), BG-Sentinel (BGS), CDC miniature light trap (CDC), mosquito drift fence (MDF), pop-up resting shelter (PRS), and the small diameter aspirator (SDA) in Florida and Panama.

	Florida				Panama			
Trap	Species Richness (s)	Simpson index (1-D)	Shannon index (H’)	Pielou index (J’)	Species Richness	Simpson Index (1-D)	Shannon Index (H’)	Pielou index (J’)
LDA	23	0.8133	2.0287	0.6470	21	0.6692	1.4998	0.5006
BGS	24	0.5722	1.3961	0.4393	7	0.7343	1.4464	0.7433
MDF	27	0.8425	2.1655	0.6570	23	0.8318	2.0335	0.6788
PRS	17	0.8120	1.8609	0.6568	18	0.6449	1.5234	0.5377
SDA	15	0.8126	1.9483	0.7194	22	0.7902	1.9450	0.6493
CDC	28	0.8333	2.0579	0.6176	19	0.7809	1.8193	0.6421

**Fig 6:**
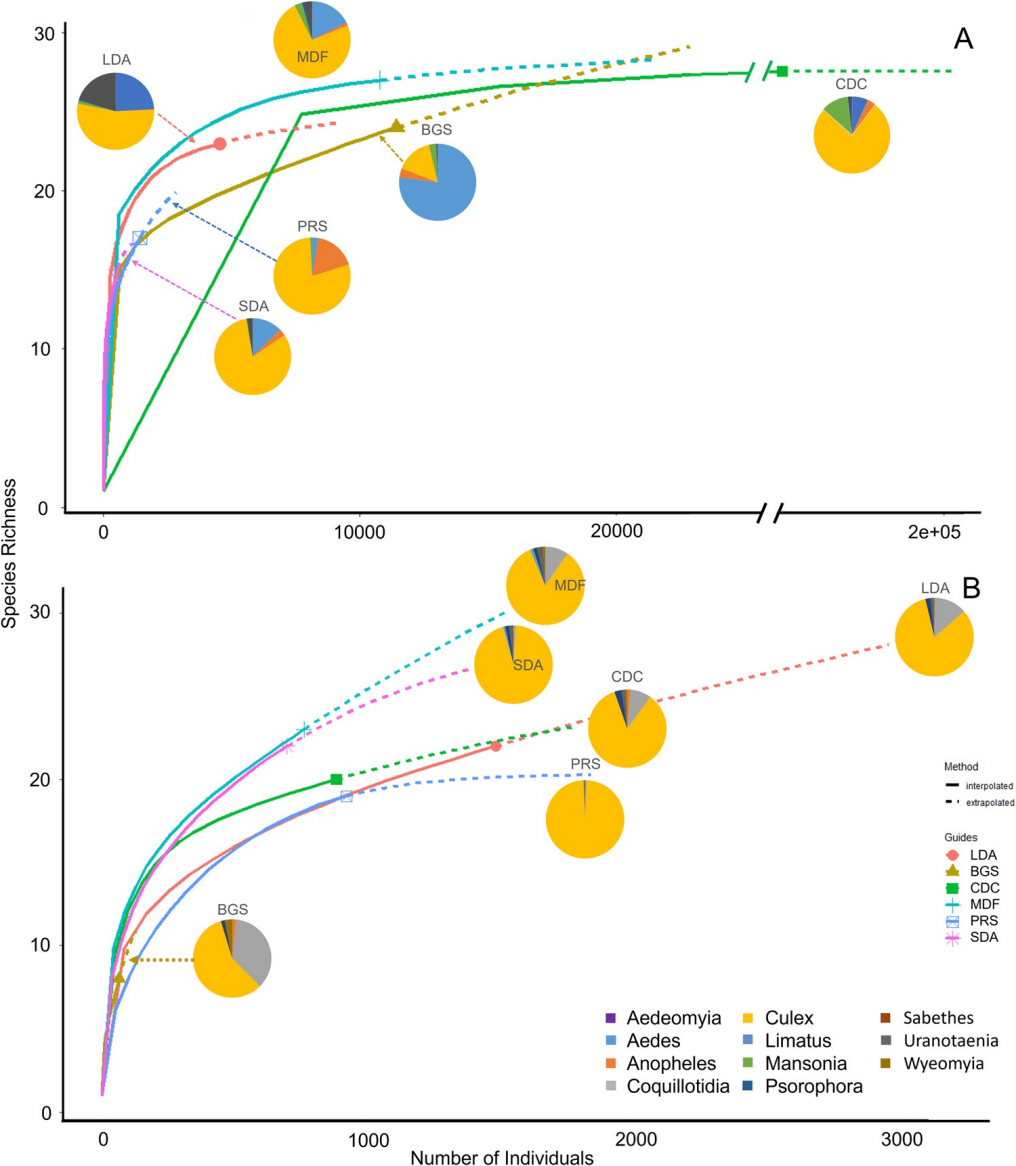
Species accumulation curves of mosquito species collected by the large diameter aspirator (LDA), BG-Sentinel (BGS), CDC miniature light trap (CDC), mosquito drift fence (MDF), pop-up resting shelter (PRS), and the small diameter aspirator (SDA) in Florida and Panama. Species accumulation curves of mosquito species captured by the six traps used in Florida City, Florida (A), and Darién, Panama (B). Pie charts correspond to the distribution of mosquito genera captured in each trap.

Rarefaction curves indicate that novel and commercial traps have different potentials for collecting diverse samples of mosquitoes in Florida (Fig 6). Species richness for each trap type varied between 15 and 28 mosquito species, despite a modest sampling effort (<15,000) for five of the six traps. The CDC trap baited with CO_2_ and MDF had similar species richness (28 and 27 respectively). Although the sampling effort for the CDC trap was nearly 13 times that of the MDF, the model extrapolates that the MDF has the capability to capture a greater number of species. The curve for the MDF reached an asymptote, and therefore sampling saturation, at around 10,000 individuals, while it estimated that over 200,000 individuals would need to be sampled for the CDC trap to reach saturation (Fig 6). The LDA was also projected to reach sampling saturation at 23 species with a slightly higher sampling effort than either the PRS or the SDA. The PRS and SDA were not predicted to reach sampling saturation given the sampling effort in the study and collected a much lower number of species than all other traps in the study (17 and 15 respectively). The diversity of mosquitoes captured by each trap was apparent by observing the dominant genera collected, for example, the genus *Culex* made up 73.7% of the MDF, 79.1% of the PRS, 81.7% of the SDA, and 76.3% of the CDC trap catches, but only 15.5% of the BGS. The two commercial traps (CDC trap (n = 138,837), and BGS (n = 11,443)), had the two highest sampling efforts of all the traps used in the study (Table 1), and the species richness was 28 and 24 respectively. The sampling effort of the drift fence (n = 10,798) was only marginally lower than the BGS (Table 1).

Rarefaction curves estimate that most traps approached the total richness across traps used in Panama. Five of the six traps did not become asymptotic given the sampling effort, suggesting that additional sampling would result in capturing additional species. Only the PRS appeared to become asymptotic and therefore reached its maximum collection potential. The SDA and MDF collected a similar species richness (22 and 23 respectively) and had a similar sampling effort (n = 695 and n = 759, respectively) (Table 2). The model extrapolated that the MDF had the capability to capture the highest number of species (Fig 6). *Culex* spp. made up between 57.8 and 98.6% of the catch for all traps (Tables 1 and 2) in Florida and Panama, respectively. The LDA had the largest sampling effort but did not reach sampling saturation.

Both the LDA and SDA varied in the diversity and assemblage of mosquito species sampled between the two countries in the study. The SDA collected a low diversity of species in Florida according to the measured diversity metrics (third lowest for both the Simpson and Shannon indices), and lowest species richness of all the traps, although evenness was highest for this trap. In contrast it had the second highest diversity for both indices in Panama, including the second highest species richness (22 species). Similarly, the LDA captured the third greatest diversity of species for both indices in Florida, species diversity and evenness collected with this trap was relatively low in Panama with diversity metrics higher than only the PRS and the BGS using Shannon’s diversity index (Table 3).

## Discussion

We used a combination of passive and active, commercially available and novel sampling devices to collect enzootic VEEV vectors in both Florida, USA and Darién, Panama. Our findings emphasize the importance of selecting trapping strategies that are appropriate and effective for the vector species and physiological state targeted by the study objectives. We found that traps varied in their utility based on geographic area, and while some traps, such as the MDF, were useful for collecting a high diversity of species, others, such as the PRS, were useful only for collecting specific groups, particularly species of *Anopheles* and *Culex*. Further, while most traps collected some blood-engorged VEEV vectors, the resting shelters were most effective for both *Cx*. *cedecei* and *Cx*. *spissipes*. The LDA was most effective for collecting blood-engorged *Cx*. *panocossa*, and the MDF was most effective for collecting *Cx*. *pedroi*. These results demonstrate that effective traps can be constructed from common materials to collect mosquitoes for VEEV vector studies and could be assessed for their utilization with vectors of other systems as well. Unfortunately, no single method was effective for capturing all species and physiological states, highlighting a particular need for assessing trap utility for the target species and physiological states of a study.

The finding that the proportions of unfed and blood-engorged mosquitoes collected by traps differed both among and between species has implications for how studies of VEEV vectors should be carried out in future investigations. Non-engorged *Cx*. *cedecei* and *Cx*. *panocossa* were efficiently collected using CDC traps, however blood-engorged specimens were mainly collected using the PRS and LDA, respectively (Fig 4). Previously, PRSs have been used to collect blood-engorged *Cx*. *cedecei* for host-use studies [28,54,55], we observed similar results during the present study, as the greatest proportion of blood-engorged *Cx*. *cedecei* were collected using the PRS. With the discovery of *Cx*. *panocossa* establishment in peninsular Florida [17], the PRS were initially used to sample blood-engorged individuals for blood meal analysis, however no blood-engorged individuals were collected using this method. In this study, few blood-fed female *Cx*. *panocossa* were collected by CDC traps, and these were primarily partially-engorged. In hindsight, this is unsurprising as over the duration of the present study only four of the 37,539 *Cx*. *panocossa* individuals were captured using PRSs, none of them blood-engorged (Table 1 and Fig 4). This study however showed that the LDA was the most efficient sampling method for collecting blood-engorged *Cx*. *panocossa*. These results suggest that after blood-feeding, *Cx*. *panocossa* tends to select resting sites in vegetation or the forest floor over cavities, in contrast to *Cx*. *cedecei*.

Interestingly, we found differences in diversity and evenness metrics between some trap types in Panama and Florida, USA, which are likely influenced by species composition in the broader region. The SDA collected the lowest species richness of mosquitoes in Florida but was relatively even in the distribution of species collected, meaning that no one species was over-represented in the collections (Table 3). In contrast, the SDA collected the second highest diversity and species richness across traps used in Panama (Table 3). Due to the size of the opening for the SDA, sampling efforts focus mainly on smaller resting areas such as tree buttresses, mouths of animal burrows, tree-holes, and stream banks that would be difficult to access with a wider diameter aspirator barrel. Resting sites such as these primarily attract *Culex*, especially subgenus *Melanoconion*, and *Anopheles* species. The diversity and richness of *Culex* (*Melanoconion*) species reaches its peak in Panama, with 63 of the 167 *Melanoconion* species found in the country [3,56–58], and this may explain why the diversity of species collected using this method was so much higher in Darién than in Florida, where there are only seven known *Melanoconion* species [17,34]. Interestingly, the SDA did not collect many Spissipes section mosquitos in Florida, (n = 12 *Cx*. *panocossa* and n = 14 *Cx*. *cedecei*, respectively), instead, these collections in Florida were dominated by Melanoconion section mosquitoes *Cx*. *pilosus* and *Cx*. *atratus*, *Culex* (*Culex*) *nigripalpus* Theobald, and *Aedes pertinax* Grabham (Table 1).

The BGS collected primarily *Aedes* spp. in Florida (Fig 6) and captured low numbers of total females (Table 2) and species (Fig 6) in Darién. Given that only three *Aedes* females (one each of *Aedes fulvus* Ross, *Aedes taeniorhynchus* Wiedemann, and *Aedes euplocamus* Dyar & Knab) were collected during the entire study period in Darién (Table 2), and considering the BGS was designed to collect anthropophagic *Aedes* species [59], these results are unsurprising. While the BGS in Florida collected a larger number of *Cx*. *cedecei* and *Cx*. *erraticus* than we anticipated (Table 1), total numbers for both species were fewer than for CDC light traps or PRSs (Table 1). It is not likely that the type of lure used would greatly affect the number of *Cx*. *erraticus* or *Cx*. *cedecei* collected with the BGS, as different lures have been shown to impact the number of *Aedes* species collected [60] but not *Culex* species [61]. Despite the demonstrated utility of the BGS trap in urban and peri-urban environments, we conclude that the BGS is not a particularly effective tool for collecting diverse zoonotic Alphavirus vectors in the American Tropics.

This study has several limitations that impact our ability to draw robust conclusions, including variations in duration of sampling periods, meteorological conditions, mosquito identifications, and subsampling between the two locations. A major inconsistency was the shorter sampling period for Darién (2 weeks) versus in Florida (6 months). In Panama, sampling was conducted during the wet season, and the mosquito community of the two-week sampling period is likely not representative of the community during the dry season. Still, our data from Darién provide an important first glimpse of how several sampling methods compare in an understudied area. As in other studies, we were unable to identify many Panamanian Melanoconion section mosquitoes to species (Table 2), potentially affecting metrics of the mosquito assemblage (species richness, evenness, diversity). It is likely that at least ten species of the Melanoconion section were present in PRS, SDA and LDA aspirator collections, given the much larger numbers of unidentifiable females present in these traps (Table 2). Interestingly, a cryptic *Cx*. *pedroi*-like species was identified in 2004, indicating that *Cx*. *pedroi s*.*l*. may represent a group of morphologically cryptic species [62] which would also influence diversity metrics of our study. Finally, subsampling likely negatively affected species richness and diversity estimates for CDC CO_2_-baited traps in Florida. While these issues likely impacted diversity estimates in both locations, our primary objective, to determine effective trapping methods for Spissipes section mosquitoes should withstand scrutiny. Our study provides valuable information on optimal sampling techniques for active and resting mosquitoes, which will assist and inform researchers in their field studies on Spissipes section vectors.

The MDF consistently collected the highest diversity of mosquitoes in the study (Table 3), suggesting that similar passive collection methods could be the best method for accurately estimating mosquito community and physiological composition in an area. Passive mosquito collection methods include both those that sample resting mosquito populations, as well as those which sample mosquitoes moving through the environment in search of blood or sugar meals, resting sites, or mates [1,63]. Burkot et al. [64] collected 377 blood-engorged mosquitoes in Indonesia, the Solomon Islands and Papua New Guinea using a barrier screen method to intercept exophilic *Anopheles* species. Pollard et al. [63] then used a similar design to the MDF and barrier screen method to collect *Anopheles*, *Culex*, and *Aedes* mosquitoes in Australia, but did not collect any blood-engorged mosquitoes. However, we collected blood-engorged females of several species using the MDF in Florida and Darién, with the MDF being the most effective trap for capturing blood-engorged *Cx*. *pedroi* in Darién, in addition to collecting blood-engorged *Cx*. *cedecei* and *Cx*. *panocossa* to a lesser extent than other traps (Fig 4).

In summary, this work provides comparative data on sampling methods for four Spissipes section mosquitoes which are vectors of enzootic VEEV subtypes, as well as characterizing the utility of different traps for capturing a wide diversity of mosquito species. In Darién, where endemic VEEV-ID transmission has occurred repeatedly over the past several years [23,31,65,66], effective sampling techniques for blood-engorged females of *Cx*. *pedroi* and *Cx*. *spissipes*, were the MDF and the PRS respectively. In Florida, effective sampling techniques included the LDA for blood-engorged *Cx*. *panocossa* and PRS for blood-engorged *Cx*. *cedecei*. The MDF was an effective trap for quantifying the mosquito assemblage without the use of attractive baits. Evaluating commercially available and novel sampling tools in a comparative field study provides information useful to designing future field studies, including studies on the host associations of *Cx*. *panocossa* in its introduced range.

## Supporting information

S1 Fig Photographs of authors KS and MS alongside all traps from the manuscript being used in the field. (PDF)

## References

[1] Silver JB. Mosquito Ecology: Field Sampling Methods. 3rd ed. Springer Netherlands; 2008. doi: 10.1007/978-1-4020-6666-5

[2] WHO. Arboviruses and human disease: Report of a WHO scientific group. World Health Organization Technical Report Series, no. 369. Geneva: World Health Organization; 1967. 4963041

[3] Sallum MA, Forattini OP. Revision of the Spissipes section of *Culex* (*Melanoconion*) (Diptera:Culicidae). J Am Mosq Control Assoc. 1996;12: 517–600. 8887711

[4] Pisano MB, Spinsanti LI, Díaz LA, Farías AA, Almirón WR, Ré VE, et al. First detection of Rio Negro virus (Venezuelan equine encephalitis complex subtype VI) in Córdoba, Argentina. Mem Inst Oswaldo Cruz. 2012;107: 125–128. doi: 10.1590/s0074-02762012000100017 22310545

[5] Monath TP, Lazuick JS, Cropp CB, Rush WA, Calisher CH, Kinney RM, et al. Recovery of Tonate virus (“Bijou Bridge” strain), a member of the Venezuelan equine encephalomyelitis virus complex, from cliff swallow nest bugs (*Oeciacus vicarius*) and nestling birds in North America. Am J Trop Med Hyg. 1980;29: 969–983. doi: 10.4269/ajtmh.1980.29.969 7435797

[6] Oberste MS, Ludwig V, Kondig JF, Weaver SC, Smith JF, Rico-hesse R. Association of Venezuelan equine encephalitis virus subtype IE with two equine epizootics in Mexico. 1998;59: 100–107. 10.4269/ajtmh.1998.59.100 9684636

[7] Forrester NL, Wertheim JO, Dugan VG, Auguste AJ, Lin D, Adams AP, et al. Evolution and spread of Venezuelan equine encephalitis complex alphavirus in the Americas. PLoS Negl Trop Dis. 2017;11: e0005693. doi: 10.1371/journal.pntd.0005693 28771475 PMC5557581

[8] Weaver SC, Ferro C, Barrera R, Boshell J, Navarro J-C. Venezuelan equine encephalitis. Annu Rev Entomol. 2004;49: 141–174. doi: 10.1146/annurev.ento.49.061802.123422 14651460

[9] Ferro C, Boshell J, Moncayo AC, Gonzalez M, Ahumada ML, Kang W, et al. Natural enzootic vectors of Venezuelan equine encephalitis virus in the Magdalena Valley, Colombia. Emerg Infect Dis. 2003;9: 49–54. doi: 10.3201/eid0901.020136 12533281 PMC2873762

[10] Weaver SC, Scherer WF, Taylor CA, Castello DA, Cupp EW. Laboratory vector competence of *Culex* (*Melanoconion*) *cedecei* for sympatric and allopatric Venezuelan equine encephalomyelitis viruses. Am J Trop Med Hyg. 1986;35: 619–623. doi: 10.4269/ajtmh.1986.35.619 3706626

[11] Cupp EW, Scherer WF, Ordonez JV. Transmission of Venezuelan encephalitis virus by naturally infected *Culex* (*Melanoconion*) *opisthopus*. Am J Trop Med Hyg. 1979;28: 1060–1063. doi: 10.4269/ajtmh.1979.28.1060 507283

[12] Galindo P, Grayson MA. *Culex* (*Melanoconion*) *aikenii*: Natural vector in Panama of endemic Venezuelan encephalitis. Science. 1971;172: 594–595. doi: 10.1126/science.172.3983.594 5555082

[13] Turell MJ. Vector competence of three Venezuelan mosquitoes (Diptera: Culicidae) for an epizootic IC strain of Venezuelan equine encephalitis virus. J Med Entomol. 1999;36: 407–409. doi: 10.1093/jmedent/36.4.407 10467764

[14] Turell MJ, Jones JW, Sardelis MR, Dohm DJ, Coleman RE, Watts DM, et al. Vector competence of Peruvian mosquitoes (Diptera: Culicidae) for epizootic and enzootic strains of Venezuelan equine encephalomyelitis virus. J Med Entomol. 2000;37: 835–839. doi: 10.1603/0022-2585-37.6.835 11126537

[15] Turell MJ, O’guinn ML, Jones JW, Sardelis MR, Dohm DJ, DM Watts, et al. Isolation of viruses from mosquitoes (Diptera: Culicidae) collected in the Amazon basin region of Peru. J Med Entomol. 2005;42: 891–898. doi: 10.1603/0022-2585(2005)042[0891:IOVFMD]2.0.CO;2 16366001

[16] Turell MJ, Dohm DJ, Fernandez R, Calampa C, O’Guinn ML. Vector competence of Peruvian mosquitoes (Diptera: Culicidae) for a subtype IIIC virus in the Venezuelan equine encephalomyelitis complex isolated from mosquitoes captured in Peru. J Am Mosq Control Assoc. 2006;22: 70–75. doi: 10.2987/8756-971X(2006)22[70:VCOPMD]2.0.CO;2 16646325

[17] Blosser EM, Burkett-Cadena ND. *Culex* (*Melanoconion*) *panocossa* from peninsular Florida, USA. Acta Trop. 2017;167: 59–63. doi: 10.1016/j.actatropica.2016.12.024 28012907

[18] Torres R, Samudio R, Carrera J-P, Young J, Márquez R, Hurtado L, et al. Enzootic mosquito vector species at equine encephalitis transmission foci in the República de Panamá. PLoS ONE. 2017;12: e0185491. doi: 10.1371/journal.pone.0185491 28937995 PMC5609755

[19] Christensen HA, Vasquez AM de, Mendez E. Patrones alimentarios de *Culex ocossa* y *Cx*. *panocossa* (Diptera, Culicidae), vectores de la encephalitis equina Venezolana en Panama. Notas Veterinarias. 1993;3: 4–8.

[20] Forattini OP, Gomes A de C, Kakitani I, Marucci D. Observações sobre domiciliação de mosquitos *Culex* (*Melanoconion*), em ambiente com acentuadas modificações antrópicas. Rev Saúde Pública. 1991;25: 257–266. doi: 10.1590/s0034-89101991000400004 1820613

[21] Galindo P, Adames AJ. Ecological profile of *Culex* (*Melanoconion*) *aikenii* (Diptera: Culicidae), vector of endemic Venezuelan encephalitis in Panama. Environ Entomol. 1973;2: 81–86. doi: 10.1093/ee/2.1.81

[22] Tempelis CH, Galindo P. Host-feeding patterns of *Culex* (*Melanoconion*) and *Culex* (*Aedinus*) mosquitoes collected in Panama. J Med Entomol. 1975;12: 205–209. doi: 10.1093/jmedent/12.2.205 1159744

[23] Carrera J-P, Cucunubá ZM, Neira K, Lambert B, Pittí Y, Liscano J, et al. Endemic and epidemic human Alphavirus infections in eastern Panama: An analysis of population-based cross-sectional surveys. Am J Trop Med Hyg. 2020;103: 2429–2437. doi: 10.4269/ajtmh.20-0408 33124532 PMC7695115

[24] Davies JB. Attraction of *Culex portesi* Senevet & Abonnenc and *Culex taeniopus* Dyar & Knab (Diptera: Culicidae) to 20 animal species exposed in a Trinidad forest. I. Baits ranked by numbers of mosquitoes caught and engorged. Bull Entomol Res. 1978;68: 707–719. doi: 10.1017/S0007485300009664

[25] Yanoviak SP, Aguilar PV, Lounibos LP, Weaver SC. Transmission of a Venezuelan equine encephalitis complex Alphavirus by *Culex* (*Melanoconion*) *gnomatos* (Diptera: Culicidae) in northeastern Peru. J Med Entomol. 2005;42: 404–408. doi: 10.1093/jmedent/42.3.404 15962794

[26] Coffey LL, Carrara A-S, Paessler S, Haynie ML, Bradley RD, Tesh RB, et al. Experimental Everglades virus infection of cotton rats (*Sigmodon hispidus*). Emerg Infect Dis. 2004;10: 2182–2188. doi: 10.3201/eid1012.040442 15663857 PMC3323382

[27] Christensen HA, Vasquez AM de, Boreham MM. Host-feeding patterns of mosquitoes (Diptera: Culicidae) from central Panama. Am J Trop Med Hyg. 1996;55: 202–208. doi: 10.4269/ajtmh.1996.55.202 8780461

[28] Edman JD. Host-feeding patterns of Florida mosquitoes (Diptera: Culicidae) VI. *Culex* (*Melanoconion*). J Med Entomol. 1979;15: 521–525. doi: 10.1093/jmedent/15.5-6.521 544824

[29] Burkett-Cadena ND, Hoyer I, Blosser E, Reeves L. Human-powered pop-up resting shelter for sampling cavity-resting mosquitoes. Acta Trop. 2019;190: 288–292. doi: 10.1016/j.actatropica.2018.12.002 30521803

[30] Powers AM, Oberste MS, Brault AC, Rico-Hesse R, Schmura SM, Smith JF, et al. Repeated emergence of epidemic/epizootic Venezuelan equine encephalitis from a single genotype of enzootic subtype ID virus. J Virol. 1997;71: 6697–6705. doi: 10.1128/JVI.71.9.6697-6705.1997 9261393 PMC191949

[31] Vittor AY, Armien B, Gonzalez P, Carrera J-P, Dominguez C, Valderrama A, et al. Epidemiology of emergent Madariaga encephalitis in a region with endemic Venezuelan equine encephalitis: Initial host studies and human cross-sectional study in Darien, Panama. PLOS Negl Trop Dis. 2016;10: e0004554. doi: 10.1371/journal.pntd.0004554 27101567 PMC4839771

[32] Willson JD, Gibbons JW. Drift fences, coverboards, and other traps. Amphibian ecology and conservation: A handbook of techniques. 2010; 229–245.

[33] Nasci R. A lightweight battery-powered aspirator for collecting resting mosquitoes in the field. Mosq News. 1981;41: 808–811.

[34] Darsie RF Jr, Ward RA. Identification and geographical distribution of the mosquitoes of North America, north of Mexico. Walter Reed Army Institute of Research Washington DC; 2004.

[35] Burkett-Cadena ND. Mosquitoes of the southeastern United States. University of Alabama Press; 2013.

[36] Williams MR, Savage HM. Identification of *Culex* (*Melanoconion*) species of the United States using female cibarial armature (Diptera: Culicidae). J Med Entomol. 2009;46: 745–752. doi: 10.1603/033.046.0404 19645276

[37] Lane J. Neotropical Culicidae. Volumes I & II. Neotropical Culicidae Volumes I & II. 1953. Available: https://www.cabdirect.org/cabdirect/abstract/19552901467

[38] Darsie RF Jr. A revised checklist of the mosquitoes of Guatemala including a new country record, *Psorophora cyanescens*. Mosq News. 1994;10: 511–514. 7707056

[39] Wilkerson RC, Strickman D, Litwak TR. Illustrated key to the female anopheline mosquitoes of Central America and Mexico. J Am Mosq Control Assoc. 1990;6: 7–34. 2324726

[40] Sirivanakarn S. A review of the systematics and a proposed scheme of internal classification of the New World subgenus *Melanoconion* of Culex (Diptera, Culicidae). Mosq Syst. 1982;14 265–333.

[41] Bram RA. Classification of *Culex* subgenus *Culex* in the new world (Diptera: Culicidae). Proc U S Natl Mus. 1967.

[42] Strickman D. *Culex pseudostigmatosoma*, *Cx*. *yojoae*, and *Cx*. *aquarius*: New Central American species in the subgenus *Culex* (Diptera: Culicidae). Mosq Syst. 1989;21: 143–177.

[43] Berlin OGW, Belkin JN. Mosquito studies (Diptera, Culicidae) XXXVI. Subgenera *Aedinus*; *Tinolestes*; and *Anoedioporpa*; of *Culex*; *Contributions of the American Entomological Institute*. Contributions of the American Entomological Institute. 1980;17: ii 1–104.

[44] Harbach RE, Peyton EL. A new subgenus of *Culex* in the Neotropical region (Diptera: Culicidae). Walter Reed Army Institute of Research, Washington, DC; 1992.

[45] Galindo P, Blanton FS, Peyton EL. A revision of the *Uranotaenia* of Panama with notes on other American species of the genus (Diptera, Culicidae). Ann Entomol Soc. 1954;47: 107–177. doi: 10.1093/aesa/47.1.107

[46] Zavortink TJ. A reclassification of the Sabethine genus *Trichoprosopon*. Mosq Syst. 1979;11: 255–257.

[47] Harbach RE. A new subgenus of the genus *Sabethes* (Diptera: Culicidae). Walter Reed Army Institute of Research, Washington, DC; 1991.

[48] Harbach RE, Petersen JL. Two species previously confused under the concept of *Sabethes tarsopus* in Central America (Diptera: Culicidae). Walter Reed Army Institute of Research, Washington, DC;1992.

[49] Harbach RE, Howard M. *Sabethes* (*Peytonulus*) *paradoxus*, a new species of Sabethini. Proc Entomol Soc Wash. 2002;104: 363–372.

[50] R Development Core Team. R: A Language and Environment for Statistical Computing. Vienna, Austria: R Foundation for Statistical Computing. 2019.

[51] Dormann CF, Fruend J, Gruber B, Dormann MCF, LazyData T, ByteCompile T. Package ‘bipartite.’ 2021.

[52] Chao A, Gotelli NJ, Hsieh TC, Sander EL, Ma KH, Colwell RK, et al. Rarefaction and extrapolation with Hill numbers: A framework for sampling and estimation in species diversity studies. Ecol Monogr. 2014;84: 45–67. doi: 10.1890/13-0133.1

[53] Sloyer KE, Burkett-Cadena ND. Development and field evaluation of a motion sensor activated suction trap to study vector-host interactions. Methods Ecol Evol. 2021;12: 204–211. doi: 10.1111/2041-210X.13500

[54] Hoyer IJ, Blosser EM, Acevedo C, Thompson AC, Reeves LE, Burkett-Cadena ND. Mammal decline, linked to invasive Burmese python, shifts host use of vector mosquito towards reservoir hosts of a zoonotic disease. Biol Lett. 2017;13: 20170353. doi: 10.1098/rsbl.2017.0353 28978755 PMC5665769

[55] Hoyer IJ, Acevedo C, Wiggins K, Alto BW, Burkett-Cadena ND. Patterns of abundance, host use, and Everglades virus infection in *Culex* (*Melanoconion*) *cedecei* mosquitoes, Florida, USA. Emerg Infect Dis. 2019;25: 1093–1100. doi: 10.3201/eid2506.180338 31107225 PMC6537747

[56] Sá ILR de Hutchings RSG, Hutchings RW Sallum MAM. Revision of the Atratus group of *Culex* (*Melanoconion*) (Diptera: Culicidae). Parasites Vectors. 2020;13: 269. doi: 10.1186/s13071-020-3982-x 32460878 PMC7251747

[57] Torres-Gutierrez C, Bergo ES, Emerson KJ, de Oliveira TMP, Greni S, Sallum MAM. Mitochondrial COI gene as a tool in the taxonomy of mosquitoes *Culex* subgenus *Melanoconion*. Acta Trop. 2016;164: 137–149. doi: 10.1016/j.actatropica.2016.09.007 27609637

[58] Sallum MAM, Hutchings RSG, Ferreira RLM. *Culex gnomatos* a new species of the Spissipes section of *Culex* (*Melanoconion*) (Diptera: Culicidae) from the Amazon region. 1997. Available: https://tspace.library.utoronto.ca/handle/1807/24008

[59] Kröckel U, Rose A, Eiras ÁE, Geier M. New tools for surveillance of adult yellow fever mosquitoes: Comparison of trap catches with human landing rates in an urban environment. J Am Mosq Control Assoc. 2006;22: 229–238. doi: 10.2987/8756-971X(2006)22[229:NTFSOA]2.0.CO;2 17019768

[60] Wilke ABB, Carvajal A, Medina J, Anderson M, Nieves VJ, Ramirez M, et al. Assessment of the effectiveness of BG-Sentinel traps baited with CO_2_ and BG-Lure for the surveillance of vector mosquitoes in Miami-Dade County, Florida. PLoS ONE. 2019;14: e0212688. doi: 10.1371/journal.pone.0212688 30794670 PMC6386269

[61] Farajollahi A, Kesavaraju B, Price DC, Williams GM, Healy SP, Gaugler R, et al. Field efficacy of BG-Sentinel and industry-standard traps for *Aedes albopictus* (Diptera: Culicidae) and West Nile virus surveillance. J Med Entomol. 2009;46: 919–925. doi: 10.1603/033.046.0426 19645298

[62] Navarro J-C, Weaver SC. Molecular phylogeny of the Vomerifer and Pedroi groups in the Spissipes Section of the subgenus *Culex* (*Melanoconion*). J Med Entomol. 2004;41: 575–581. doi: 10.1603/0022-2585-41.4.575 15311446

[63] Pollard EJM, Russell TL, Burkot TR. Maximising mosquito collections from barrier screens: The impacts of physical design and operation parameters. Parasites Vectors. 2019;12: 31. doi: 10.1186/s13071-019-3291-4 30642379 PMC6332603

[64] Burkot TR, Russell TL, Reimer LJ, Bugoro H, Beebe NW, Cooper RD, et al. Barrier screens: A method to sample blood-fed and host-seeking exophilic mosquitoes. Malar J. 2013;12: 49. doi: 10.1186/1475-2875-12-49 23379959 PMC3574015

[65] Aguilar PV, Estrada-Franco JG, Navarro-Lopez R, Ferro C, Haddow AD, Weaver SC. Endemic Venezuelan equine encephalitis in the Americas: Hidden under the dengue umbrella. Future Virol. 2011;6: 721–740. doi: 10.2217/FVL.11.5 21765860 PMC3134406

[66] Quiroz E, Aguilar PV, Cisneros J, Tesh RB, Weaver SC. Venezuelan equine encephalitis in Panama: Fatal endemic disease and genetic diversity of etiologic viral strains. PLoS Negl Trop Dis. 2009;3: e472. doi: 10.1371/journal.pntd.0000472 19564908 PMC2697379

