# Supplementary material for: Evaluating sampling strategies for enzootic Venezuelan equine encephalitis virus vectors in Florida and Panama
Source: PLoS Negl Trop Dis. 2022 Apr 13;16(4):e0010329. doi: 10.1371/journal.pntd.0010329 (PMC9007344; doi:10.1371/journal.pntd.0010329)
Supplement: S1 Fig — (PDF) [file pntd.0010329.s001.pdf]

Traps in field locations - Panama

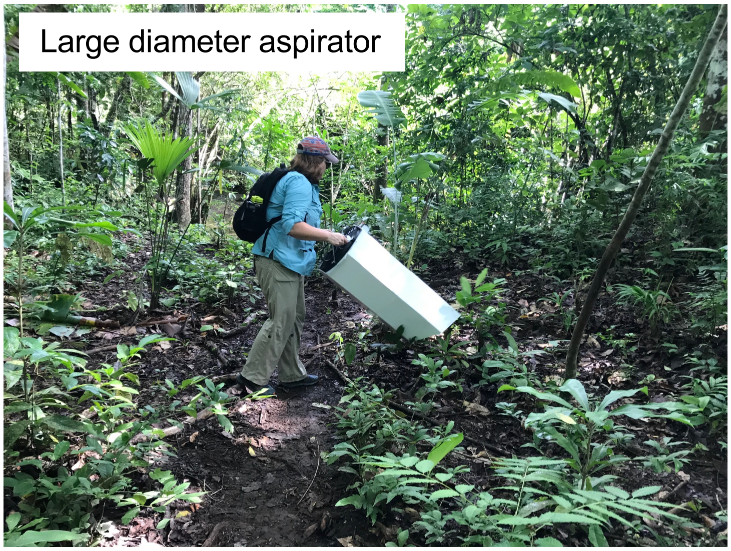
Large diameter aspirator

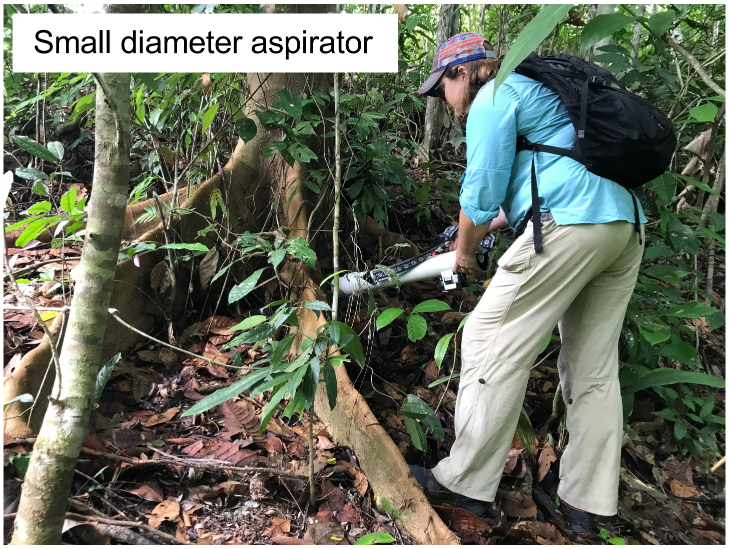
Small diameter aspirator

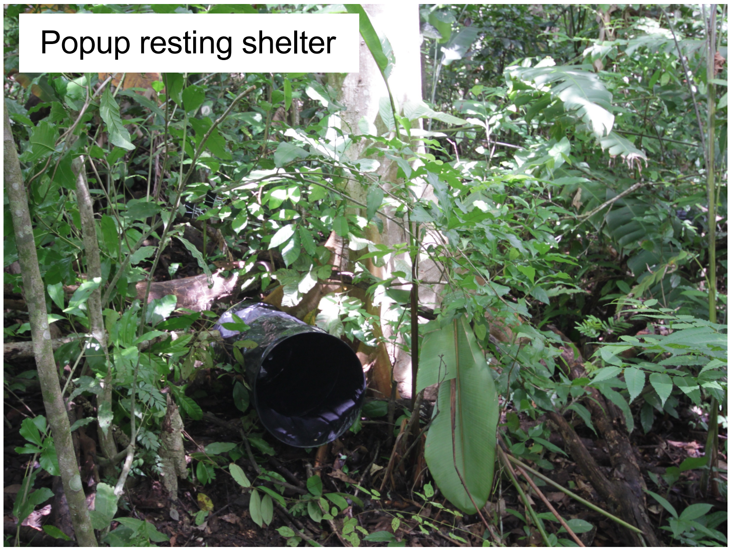
Popup resting shelter

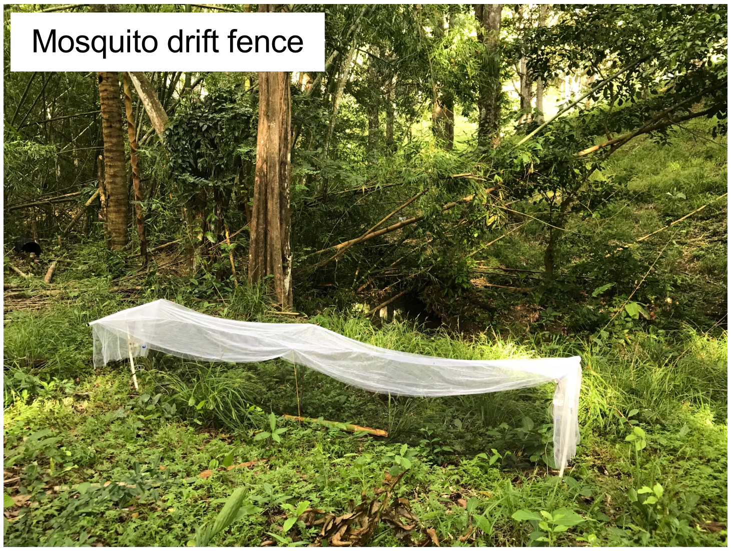
Mosquito drift fence

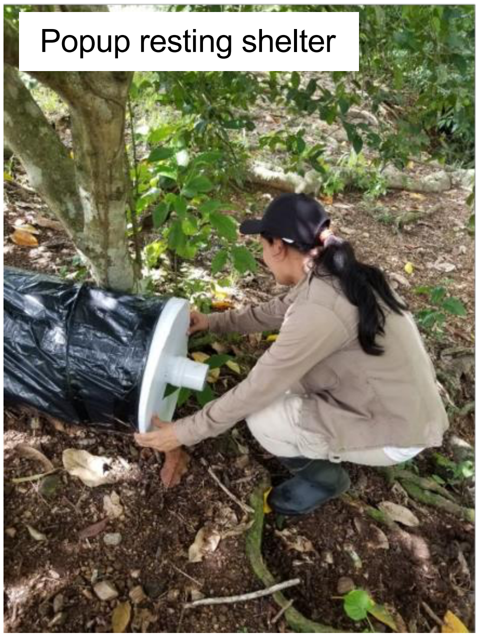
Popup resting shelter

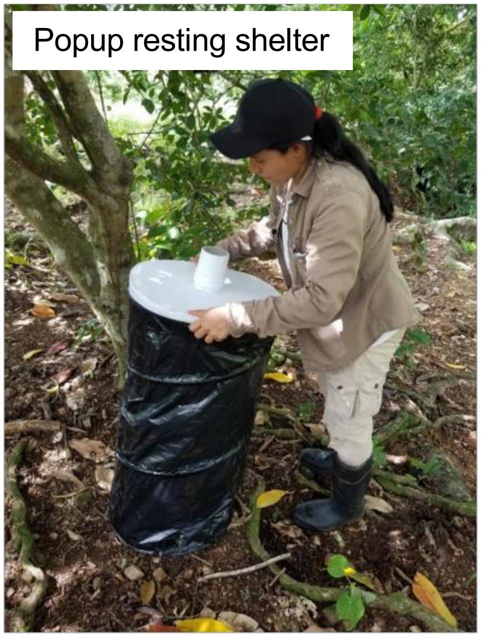
Popup resting shelter

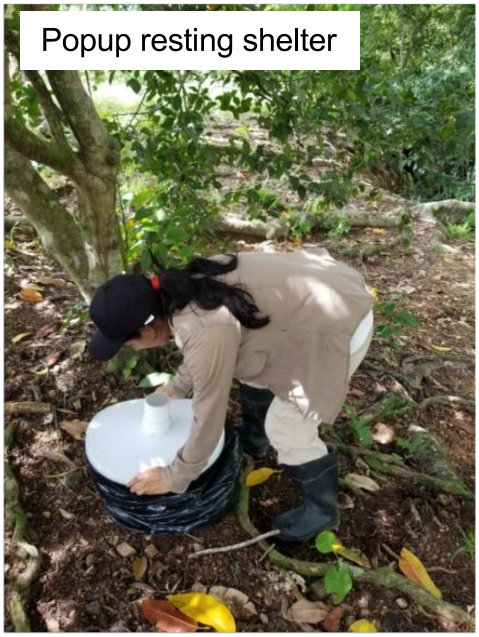
Popup resting shelter
